# Six autoantibodies associated with autoimmune encephalitis are not detectable in the cerebrospinal fluid of suicide attempters

**DOI:** 10.1371/journal.pone.0176358

**Published:** 2017-04-27

**Authors:** Johan Fernström, Åsa Westrin, Cécile Grudet, Lil Träskman-Bendz, Lena Brundin, Daniel Lindqvist

**Affiliations:** 1 Department of Clinical Sciences Lund, Psychiatry, Lund University, Lund, Sweden; 2 Psychiatric Clinic, Lund, Division of Psychiatry, Lund, Sweden; 3 Laboratory of Psychiatry and Behavioral Medicine, Center for Neurodegenerative Science, Van Andel Research Institute, Grand Rapids, Michigan, United States of America; 4 Department of Psychiatry, School of Medicine, University of California San Francisco, School of Medicine, San Francisco, California, United States of America; University of Florida, UNITED STATES

## Abstract

Previous findings suggest a link between neuroinflammatory processes and suicidality. Despite several lines of evidence supporting this link, including increased pro-inflammatory markers in blood-, cerebrospinal fluid (CSF)- and in post-mortem brain samples from suicidal individuals, the underlying mechanisms remain poorly understood. In this pilot study, we explored the possibility that autoimmune encephalopathies might be found among suicide attempters. We analysed the presence of six different autoantibodies (*N*-methyl-D-aspartate receptor, the α-amino-3-hydroxy-5-methyl-4-isoxazol-propionic acid receptor, the γ-amino-butyric acid B-receptor, the leucine-rich, glioma-inactivated 1, the contactin-associated protein-like 2, and the dipeptidyl-peptidase-like protein-6), all previously associated with psychopathology, in CSF samples from 29 unmedicated suicide attempters. Five of these subjects had high CSF/serum albumin ratio, indicative of increased blood-brain-barrier permeability. We were not able to detect any of these autoantibodies in the CSF samples. These pilot data do not support a role for autoimmune encephalopathies in suicidal behaviour, although the presence of lower levels of these autoantibodies cannot be ruled out in these patients.

## Introduction

Several lines of evidence suggest that neuroinflammatory processes are involved in the pathophysiology of suicidal behavior [1]. Immunomodulating therapies such as interferons, used to treat certain malignancies and infections, may trigger symptoms of depression and suicidality [2–4]. Suicide attempters display increased levels of pro-inflammatory markers in blood [5, 6] and cerebrospinal fluid (CSF) [7]. Moreover, impulsivity, a personality trait associated with high risk for suicidal behavior, has been associated with inflammation [8]. In further support of a link between neuroinflammation and suicide, post-mortem studies have reported microgliosis [9] and elevations of pro-inflammatory cytokine-messenger-Ribonucleic acids in the brains of suicide victims [10]. These initial findings were recently synthesized in a meta-analysis providing support for a link between suicidality and aberrant cytokine levels in blood, CSF, and postmortem brain samples [11].

The underlying pathobiology behind neuroinflammation in suicidality is not fully understood. Suggested causal mechanisms include latent infections such as *Toxoplasma gondii* [12–14], as well as allergies and asthma [15, 16]. Importantly, autoimmune mechanisms have not been well studied in relation to suicidality. Increased suicide rates have been reported in individuals with autoimmune disorders such as systemic lupus erythematosus [17], multiple sclerosis [18], and celiac disease [19]. Several lines of research over the last decade have shown that autoimmune encephalopathies may generate various types of psychiatric symptoms including psychosis, depression, confusion, agitation, and emotional instability [20, 21]. A few studies have reported detection of autoantibodies in blood samples from subjects with schizophrenia [22, 23], although not all are in agreement [24, 25]. Furthermore, increased CSF levels of immunoglobin G (IgG), with affinity for dopaminergic receptors, have been reported in suicide attempters compared to healthy controls [26]. Moreover, Kruse et al. reported that suicidal behavior was one of the symptoms presented by psychiatric inpatients seropositive for autoantibodies, and in those with *N*-methyl-D-aspartate receptor (NMDAR) IgG in CSF [27].

The etiologic mechanisms behind the generation of autoantibodies in individuals with psychiatric manifestations are not fully understood, but may involve a compromised blood-brain-barrier (BBB) [28].

In the present study we set out to detect autoantibodies in CSF samples from suicide attempters, a group often characterized by depressive symptoms, emotional instability, and even psychotic symptoms. The main purpose was to explore and develop preliminary data on the possibility that autoimmune processes may be involved in suicidal behavior. To this end, we analyzed six different autoantibodies in CSF samples from 29 suicide attempters, randomly selected from a larger cohort, employing an assay used for clinical diagnosis of autoimmune encephalitis. Within this larger cohort of suicide attempters, CSF/serum albumin ratio, an indicator of BBB integrity, has been quantified and this data have been previously published [29]. In the present study, we used CSF since it is considered to be a more sensitive medium for autoantibody detection compared to serum [27, 30].

## Methods

### Ethical approval

All parts of this study were approved by the Lund University Medical Ethics Commitee. All subjects gave verbal informed consent to participate in the study. Written informed consent was not required per the ethical approval. Consent was registered in the patients’ chart. Subjects who, in the investigator's judgment, lacked the ability to make an informed decision regarding study participation were not included in the study.

### Subject recruitment

Between 1987 and 2001, 29 subjects were enrolled in the study on admission to Lund University after a suicide attempt, as defined by Beck et al. [31]. Suicide attempters were evaluated by a certified psychiatrist for DSM axis I and II diagnoses [32]. Depressive symptoms were rated using the Montgomery-Åsberg Depression Rating Scale [33]. The 29 subjects included in this pilot study were randomly selected from a larger cohort of suicide attempters, that has been described in more detail elsewhere [7]. All subjects underwent a general physical examination before the lumbar puncture, and they were all somatically healthy except for two subjects with migraines, and two subjects with gastritis. Although some of the subjects had previously been treated with psychoactive medications, they did not receive any antidepressants or antipsychotics during a washout-period before the lumbar puncture. Occasional doses of benzodiazepines were allowed during the washout. Lumbar punctures were performed in the morning between 8 am and 9 am, after a night of fasting and bed rest. CSF was collected from the L4-L5 interspace using a standardized protocol, and immediately stored at -80°C. Twenty of the 29 CSF samples had been previously thawed/frozen at least once before the analyses.

### Assays

Six different autoantibodies previously implicated in autoimmune encephalitis [20] were analyzed; NMDAR, the α-amino-3-hydroxy-5-methyl-4-isoxazol-propionic acid receptor (AMPAR), the γ-amino-butyric acid B-receptor (GABAbR), the leucine-rich, glioma-inactivated 1 (LGI1), the contactin-associated protein-like 2 (Caspr2), and the dipeptidyl-peptidase-like protein-6 (DPPX). These autoantibodies, associated clinical syndromes and frequent symptoms are summarized in Table 1 (adapted from Wandinger et al 2011 [34]).

**Table 1:** Autoantibodies tested in the present study, associated clinical syndromes and frequent symptoms (adapted from Wandinger et al 2011).

Autoantibody	Clinical syndrome	Frequent symptoms
Anti-glutamate receptor (type NMDA)	Anti-glutamate receptor (type NMDA) encephalitis	Psychosis, memory-/language impairment, seizures, impaired consciousness, dyskinesia, movement disorders, dysautonomia, hypoventilation
Anti-glutamate receptor (type AMPA)	Limbic encephalitis, atypical psychosis	Memory deficits, confusion, disorientation, seizures, agitation, aggressive behaviour
Anti-GABAB receptor	Limbic encephalitis	Seizures, confusion, memory deficits, behavioural disorders, paranoia, hallucinations
Anti-LGI1	Limbic encephalitis	Epilectic seizures, memory deficits, confusion, dis- orientation, hyponatriaemia, myoclonus, dysautonomia
Anti-CASPR2	Neuromyotonia, Morvan‘s syndrome, Limbic encephalitis	Peripheral neuronal hyperexcitability, muscle spasms/ fasciculations/myokymia, seizures, memory deficits, confusion, disorientation, neuropathic pains, sleeping disorders, dysautonomia, weight loss
Anti-DPPX	Autoimmune encephalitis	Anxiety, forgetfulness, confusion, hallucinations, muscle spasms, tremor and pleocytosis (in CSF)

Abbreviations: NMDA: N-methyl-D-aspartate, AMPA: α-amino-3-hydroxy-5-methyl-4-isoxazole propionic acid, GABA: γ-amino butyric acid, LGI1: leucine-rich glioma-inactivated protein 1, CASPR2: contactin-associated protein 2, DPPX: dipeptidyl aminopeptidase-like protein 6

Antibody detection was done using the Autoimmune Encephalitis 6 Biochip mosaics (EUROIMMUN, Lübeck, Germany) by trained laboratory personnel at a specialized diagnostic laboratory affiliated with Lund University Hospital (Wieslab, Malmö, Sweden). Each biochip contains transfected cells expressing a specific antigen (NMDAR, AMPAR, GABAbR, LGI1, Caspr2, and DPPX). Biochip mosaics were incubated each with 30 μl of un-diluted CSF for 30 minutes at room temperature, washed with PBS-Tween, and immersed in PBS-Tween for 5 minutes. Bound antibodies were stained with fluorescein isothiocyanate–labeled goat anti-human IgG, (Euroimmun) for 30 minutes at room temperature. Slides were washed again with a flush of PBS-Tween and then immersed in PBS-Tween for 5 minutes. Drops of PBS-buffered glycerol were placed onto a cover glass, and the biochip slides were embedded in this mounting medium simultaneously and examined by fluorescence microscopy. Positive and negative controls were included with every test procedure. Autoantibodies against the corresponding antigen react specifically with the corresponding transfected cells and results are given as positive or negative. NMDA receptor autoantibodies react specifically with the corresponding transfected cells and induce a fine granular cytoplasmic fluorescence, while the cell nuclei are only slightly stained. Antibodies against AMPA receptors, CASPR2, LGI1, and GABAB1/B2 receptors react specifically with the cytoplasm of the corresponding transfected cells inducing a cytoplasmic fluorescence, with some fluorescence of the cell membrane, while the cell nuclei are only slightly stained. Antibodies against DPPX react with the transfected cells of the test substrate. They produce a spread, smooth to fine-speckled cytoplasmic fluorescence, partly with fluorescence of the cell membrane. The cell nuclei are only slightly stained.

Samples with no autoantibodies or negative control cells results in no fluorescent staining. The test results were interpreted manually by extensively trained personnel by reading the fluorescence of fixated cells in a microscopy and results are reported as positive or negative. There are no predetermined cut-off values, since a negative result will show no fluorescence.

Albumin was determined in serum and CSF as previously described [29]. CSF/serum albumin ratio was calculated as CSF albumin (g/l)/serum albumin (mg/l).

## Results and discussion

### Demographics

Demographic and clinical characteristics of the study participants are summarized in Table 2. Approximately two-thirds of the subjects had Axis II co-morbidity, cluster B being the most frequent specifier (n = 10).

**Table 2:** Demographic and clinical characteristics of study participants (n = 29).

Age (mean ± SD)	41 ± 14
Sex	13 men, 16 women
BMI (mean ± SD)	24 ± 4
Number of wash-out days (mean ± SD)	12 ± 7
MADRS score	17 ± 11
Principal Axis 1 diagnosis (n)	MDD = 8 Dysthymic disorder = 1 Adjustment disorder = 5 Depression NOS = 5 Substance use disorder = 2 Schizoaffective disorder = 1 Psychosis NOS = 1 No axis 1 disorder = 6
Axis II co-morbidity (n)	19

Abbreviations: MADS = Montgomery-Åsberg Depression Rating Scale; BMI = Body Mass Index; DSM = Diagnostic and Statistical Manual of Mental Disorders; NOS = Not Otherwise Specified

Most of the subjects (n = 21) had made a non-violent suicide attempt, e.g. intoxication, while the remainder (n = 8) had made a violent suicide attempt, e.g. carbon monoxide poisoning or jumping in front of a train [35]. Ten of the subjects had attempted suicide more than once and for 19 subjects this was the first attempt.

### Autoantibodies in CSF

None of the following autoantibodies were detected in any of the CSF samples: NMDAR, AMPAR, GABAbR, LGI1, Caspr2, DPPX.

### CSF/serum albumin ratio

Five subjects had increased CSF/serum albumin ratio, in relation to age-adjusted reference values previously described [36]. For individuals <45 years values above 6.8 were considered high, while the cut-off was 10.2 for those aged 45 and over.

### Interpretation and discussion of results

In this pilot study we investigated, the presence of autoimmune antibodies in CSF samples from a group of recent suicide attempters. Given that neuroinflammation has been implicated in the pathophysiology of suicidality [1], this is an important research question that needs to be pursued. Although classical autoimmune encephalopathies are typically associated with aberrant neurological signs and symptoms, such as seizures, affected motor functions and speech, there is also a growing interest in autoimmune encephalopathies that may present with “pure” psychiatric symptoms. The present sample was randomly selected from a larger cohort of somatically healthy and medication-free recent suicide attempters. We tested six different autoantibodies previously implicated in clinical encephalopathies characterized by a psychiatric symptoms [20]. The findings of the present study were negative; we did not detect any of these autoantibodies in any of the CSF samples. These results, however, should be interpreted with some caution as outlined below.

Various psychiatric manifestations have been documented in subjects with anti-NMDAR encephalitis and other autoimmune encephalitis. These include psychotic symptoms, cognitive impairment, depression, irritability, and personality disturbances [37, 38]. Fewer studies have investigated the presence of autoantibodies in primary psychiatric cohorts. The available studies have detected NMDAR antibodies in blood samples from subjects diagnosed with schizophrenia [22, 23], although there have also been negative studies [24, 25]. Bergquist et al showed that suicide attempters have increased CSF levels of immunoglobin G with affinity for dopaminergic receptors (DA-IgG) compared to healthy controls [26]. Comparability between that study and the present one is, however, limited since different assays were used and Bergquist et al. measured one specific antibody (DA-IgG) while we explored six different autoantibodies implicated in autoimmune encephalitis.

In those cases autoantibodies are detected in individuals with psychiatric and neurologic manifestations, their origin is not fully understood. Several potential mechanisms have been proposed including paraneoplastic processes, past influenza infections and genetic susceptibility [28, 39]. Moreover, it has been hypothesized that a compromised BBB may facilitate transportation of autoantibodies from the periphery to the brain [28]. In our sample, approximately 17% of the subjects had high CSF/serum albumin ratio, indicative of increased BBB permeability, yet CSF autoimmune antibodies were not detectable in any of these subjects. Our findings do not support the involvement of impaired BBB integrity in autoimmune processes in psychiatric patients, although larger case series are clearly needed in order to definitely confirm or refute this hypothesis.

The present study is among the first to investigate autoantibodies in CSF samples from psychiatric subjects. An important strength of the study is the inclusion of a well-characterized sample of unmedicated recent suicide attempters. The present study, however, also comes with several caveats. Firstly, the sample size was relatively small, thus any conclusions must be considered preliminary and the results should serve primarily as pilot data for testing in future larger scale studies. Even though the test panel that was used included six different autoantibodies, thus increasing the possibility of detection, the number of subjects tested might have been insufficient. Secondly, the assays used in our study were originally designed as a diagnostic tool for targeted populations of neurology patients where autoimmune processes in the brain are suspected. Thus, we cannot rule out that a more sensitive experimental assay would have been able to detect more subtle levels of autoantibodies. Future studies might consider using experimental assays where lower concentrations of autoantibodies might be detected. Finally, this is a cross-sectional study and we cannot completely rule the possibility that autoantibodies, if present, might have been detectable immediately after the suicide attempt but not after the wash-out period at the time of the lumbar puncture. Longitudinal studies of anti-NMDA receptor encephalitis, however, have shown that although titres may decrease over time, antibodies are often detected in consecutive samples [30].

In conclusion, we were not able to detect any autoantibodies in CSF samples from recent suicide attempters. This is one of the first studies of its kind, and future larger scale studies using sensitive assays are warranted before any firm conclusions may be drawn.
